# Circulating EV miRNA Cargo in Glioblastoma Patients Is Associated with Distinct Gene Expression Signatures in Peripheral Immune Cells, Suggesting an Early, Compartment-Specific Immune Priming State

**DOI:** 10.3390/biomedicines14030703

**Published:** 2026-03-18

**Authors:** Marija Popovic-Vukovic, Ivana Kolic, Aleksandra Stankovic, Maja Zivkovic, Mihailo Milicevic, Ivan Bogdanovic, Ivana Srbljak, Nina Petrovic, Tatjana Stanojkovic, Marina Nikitovic, Ivan Jovanovic

**Affiliations:** 1 Department of Radiation Oncology, Institute of Oncology and Radiology of Serbia, Pasterova 14, 11000 Belgrade, Serbia; maja1083@gmail.com (M.P.-V.); marina.nikitovic@ncrc.ac.rs (M.N.); 2 Faculty of Medicine, University of Belgrade, Dr Subotica Starijeg 8, 11000 Belgrade, Serbiaivanbg83@gmail.com (I.B.); 3 Department of Radiobiology and Molecular Genetics, “Vinca” Institute of Nuclear Sciences, National Institute of the Republic of Serbia, University of Belgrade, Mike Petrovica Alasa 12-14, 11351 Belgrade, Serbia; ivanak@vin.bg.ac.rs (I.K.); alexas@vin.bg.ac.rs (A.S.); majaz@vin.bg.ac.rs (M.Z.); 4 Clinic of Neurosurgery, Neuro-Oncology Department, University Clinical Center of Serbia, Dr Koste Todorovica 4, 11000 Belgrade, Serbia; 5 Department of Experimental Oncology, Institute of Oncology and Radiology of Serbia, Pasterova 14, 11000 Belgrade, Serbia; srbljak.ivana@gmail.com (I.S.); dragoninspiration@yahoo.com (N.P.); stanojkovict@ncrc.ac.rs (T.S.)

**Keywords:** glioblastoma, miRNA, exosomes, bioinformatics, gene expression

## Abstract

**Background**: Glioblastoma is the most lethal primary brain tumor, being characterized not only by marked intratumoral heterogeneity but also by strong systemic immunosuppression. Circulating extracellular vesicles (EVs) have gained growing recognition during the past decade as important mediators of intercellular communication, particularly through their microRNA (miRNA) cargo. However, the global EV miRNA landscape of circulating EV-associated miRNAs in glioblastoma patients and their relation with gene expression patterns in peripheral immune cells remain incompletely defined. **Methods**: To investigate these systemic associations, we profiled EV-associated miRNA expression in plasma samples from glioblastoma patients and matched healthy controls using the small RNA sequencing method, followed by differential expression and pathway analyses. Based on these findings and literature evidence, identified changes in selected EV miRNA levels were validated by qPCR in an extended cohort. In parallel, expression of their predicted immune-related mRNA targets was analyzed in peripheral blood mononuclear cells (PBMCs) obtained from the same individuals, allowing for the assessment of EV miRNA–PBMC mRNA correlation patterns. **Results**: Small RNA sequencing revealed a distinct circulating EV-associated miRNA profile in glioblastoma patients compared to controls. The validation analysis of relative expression of the identified DEmiRNAs has shown a statistically significant upregulation of hsa-miR-142-3p, hsa-miR-19b-3p, and hsa-miR-98-5p in circulating EVs of glioblastoma patients compared to controls. PBMCs from glioblastoma patients exhibited increased expression of the regulatory genes *SOCS1*, *SOCS3*, and *PTEN,* while *CCND1* was downregulated. Correlation analyses suggested that certain EV miRNA changes parallel with alterations in PBMC gene expression in glioblastoma patients, suggesting early immune priming in the circulation. **Conclusions**: Our findings indicate that circulating EV miRNAs in glioblastoma patients are associated with specific gene expression patterns in peripheral immune cells, suggesting a complex regulatory balance between pro-inflammatory and anti-inflammatory cues, potentially preceding full tumor-associated macrophage polarization. These molecular interactions may offer opportunities for developing early biomarkers or new therapeutic approaches.

## 1. Introduction

Glioblastoma is the most lethal primary brain tumor, with a median survival of only ~15 months [1] and a 5-year survival below 10% [2]. Its poor prognosis stems from extensive intratumoral heterogeneity [3] and a profoundly immunosuppressive microenvironment [4]. Glioblastoma is often described as an “immunologically cold” tumor: it harbors very few cytotoxic T cells but abundant immunosuppressive myeloid cells [5,6,7,8]. Tumor-associated macrophages (TAMs) derived from both brain-resident microglia and blood monocytes dominate the immune infiltrate, comprising up to 30–50% of the tumor mass [9,10], with monocyte-derived macrophages accounting for the majority [11]. These TAMs exhibit an M2-like phenotype that promotes tumor growth and dampens anti-tumor immunity [12,13]. Although this growing body of research aims to elucidate the crosstalk mechanisms between glioblastoma, macrophages, and microglia, our understanding of how tumor-associated macrophages (TAMs) are stimulated to adopt a glioblastoma-supportive phenotype and the agents responsible for initiating these responses remains incomplete.

Beyond the tumor microenvironment, glioblastoma exerts systemic immunosuppressive effects. Extracellular vesicles (EVs), nanoscale lipid-bound particles released by a variety of cell types, including tumor and stromal cells, have emerged as potentially key mediators of this systemic influence [14]. EVs isolated from the plasma of glioblastoma patients are significantly more abundant than those from healthy controls, with levels correlating with tumor burden and rising prior to radiographic recurrence [15]. These EVs carry regulatory cargo, including microRNAs (miRNAs), that can modulate gene expression and reprogram recipient immune cells [16]. Certain EV-associated miRNAs reported in glioblastoma, including miR-21, miR-10a, and miR-27a-3p, have already been shown to drive M2 polarization of macrophages, thus contributing to an immunosuppressive milieu [17,18]. Circulating monocytes are rapidly responding to systemic cues even before tissue migration [19]; thus, their transient presence in blood makes them highly responsive to circulating signals, including EVs, which can deliver immunosuppressive miRNAs [20], priming them for tissue-level immune reprogramming in glioblastoma.

Although studies have already investigated EV miRNAs’ role in glioblastoma/macrophage/microglia crosstalk, most have been conducted using cell lines and have focused on preselected candidate miRNAs [21]. Only a limited number of studies have employed high-throughput expression profiling of circulating EV miRNAs in glioblastoma patients, and these have primarily aimed to identify circulating miRNA biomarkers. However, their findings have been inconsistent [22,23,24,25]. Importantly, no studies to date have combined comprehensive profiling of circulating EV-associated miRNAs with parallel ex vivo analysis of predicted target gene expression in peripheral blood mononuclear cells (PBMCs) within the same glioblastoma patient cohort, leaving the systemic EV-associated miRNA landscape and its association with PBMC gene expression changes incompletely characterized.

To address these gaps, we first aimed to identify and validate differentially expressed circulating EV miRNAs in glioblastoma patients compared with healthy controls. Building on this, for each patient enrolled in the study, we performed comprehensive analyses of both EV miRNAs and their predicted target gene expression in PBMCs. This approach was designed to provide complementary insights into systemic immune alterations in glioblastoma and to estimate the potential of circulating EV cargo to contribute to immune reprogramming in glioblastoma patients.

## 2. Materials and Methods

### 2.1. Subjects

The study group included 43 patients who underwent surgical intervention of diagnosed glioblastoma removal at the Clinic of Neurosurgery, University Clinical Center of Serbia, Belgrade. The parameters collected from the patients’ medical charts were demographic characteristics (age and gender), comorbidities and therapy, previous history of malignant diseases, and size and localization of the tumor identified by neuroimaging and operative data. Blood samples in disodium EDTA were obtained one day before surgery and immediately underwent further extraction of EV and PBMC total RNA. After surgery, an experienced neuropathologist reviewed all the slides and confirmed histopathological diagnosis of glioblastoma IDH-wildtype at the Department of Pathology, University Clinical Center of Serbia, Belgrade. Inclusion criterion was neuropathologically confirmed glioblastoma IDH-wildtype. Exclusion criteria were previous malignant disease, oncology treatment, and chronic inflammatory and autoimmune diseases. The glioblastoma age- and sex-matched control group comprised 47 healthy volunteers with no previous personal history of malignant and chronic disease. All participants signed a statement providing their written informed consent. The Ethical Committee of the Faculty of Medicine, University of Belgrade approved the study protocol (approval number: 1322/II-10, 20 February 2020), and the research was carried out in compliance with the Declaration of Helsinki.

### 2.2. Isolation of PBMCs and Extraction of the Total RNA

The PBMCs were isolated from the peripheral blood samples using lymphocyte separation medium (PAA, GE Healthcare, Chicago, IL, USA), and the total RNA was extracted from PBMCs by using TRI Reagent^®^ (Ambion, Life Technologies, Austin, TX, USA). Extracted total RNA samples were dissolved in nuclease-free water (Ambion, Life Technologies, Austin, TX, USA) and stored at −80 °C. Total RNA concentration and purity were determined with a NanoDrop ND-1000 spectrophotometer (Thermo Scientific, Waltham, MA, USA).

### 2.3. Isolation and Characterization of Plasma EVs and Extraction of the Total RNA

Plasma samples (V = 1 mL) were used for the isolation of circulating extracellular vesicles, representing a pool of vesicles from diverse cell types, including tumor-derived vesicles, using the Plasma/Serum Exosome Purification Mini Kit protocol. (NorgenBiotek, Thorold, ON, Canada). Total RNA extraction from equal volumes (200 µL) of purified EVs was performed using the Exosomal RNA Isolation Kit (NorgenBiotek, Thorold, ON, Canada). The extracted RNA and the excess volume of purified EVs were stored at −80 °C until further analysis.

Nanoparticle tracking analysis (NTA) was used to analyze the concentration and size distribution of total particles in random control samples using remaining purified EVs, on a ZetaViewQuatt PMX-430 instrument with ZetaView software version 8.05.16 SP3 (Particle Metrix, Inning, Germany). The instrument was set by performing automatic cell check, camera and laser alignment, and focus optimization, using 100 nm polystyrene beads, according to the manufacturer’s instructions. Samples were diluted in PBS to obtain optimal particle count per frame (50–200). For analysis in scatter mode, a blue laser (488 nm) was used, and the video was captured at a sensitivity of 78, a shutter speed of 100, and a frame rate of 30 frames per second, in one cycle. Post-acquisition parameters were set to minimal area 10, maximal area 1000, and minimum brightness 25.

Control EV samples were further processed using the Application Kit™: EV Profiler 2 (Cat. no. 900-00079, ONI UK, Oxford, UK) and imaged using direct Stochastic Optical Reconstruction Microscopy (dSTORM) on the Nanoimager S Mark III microscope (ONI, Oxford, UK) by a technical expert from ONI. EV samples were affinity captured onto a 4-lane chip using biotinylated Tetraspanin Trio capture (anti-CD81/anti-CD63/anti-CD9). EVs were fixed for 10 min before labeling with anti-CD81-647, anti-CD63-561, and anti-CD9-488 conjugated to dSTORM-compatible dyes provided in the kit. EV samples were imaged on the Nanoimager using a 1.45 100× oil immersion objective. The system was calibrated using a bead slide before imaging the samples. TIRF illumination was used at an angle of 54.1°. Images were acquired at 30 ms exposure, and 1000 frames per channel were collected. A total of 6 fields of view (FOVs) were recorded per sample. Microscopy images were processed using ONI’s online platform CODI (alto.codi.bio) by applying drift correction and dSTORM filtering.

### 2.4. Small RNA-Seq Library Synthesis from Total RNA of Circulating EVs Samples

Synthesis of small RNA libraries was performed according to the SMARTer smRNA-Seq Kit for Illumina protocol (Takara Bio, Shiga, Japan) on the circulating EVs’ RNA samples of randomly selected six glioblastoma patients and six age- and sex-matched healthy control subjects. In brief, 7 µL of total RNA underwent a polyadenylation step followed by the cDNA synthesis. Regarding control RNA, miR163s was used as a positive control for successful library synthesis; a no-RNA negative control was also included to supervise for possible contamination due to relatively high number of PCR cycles during library production (17 cycles of PCR cDNA amplification). Indexing strategy was planned according to the Illumina TruSeq^®^ CD dual index pooling guide (Illumina Index Adapters Pooling Guide). PCR products were purified using the NucleoSpin Gel and PCR Clean-Up kit (MACHEREY-NAGEL, Düren, Germany). Library validation for both groups, as well as positive and negative controls, was conducted before the size-selection procedure using a Qubit 3 Fluorometer and Qubit dsDNA HS Assay Kit. After validation of successful library synthesis based on sufficient library concentration, library size selection was performed using AMPure XP Beads reagent (Beckman Coulter, Brea, CA, USA). Following multiple steps of size selection, the libraries are resuspended in Tris Buffer and stored at −20 °C until sequencing. The molarity of the libraries was calculated according to the Illumina Nanomolar Conversion calculator (https://support.illumina.com/help/nanomolar-conversion/nanomolar-conversion.htm, accessed on 15 January 2026) using 300 bp as the average library size. Libraries were diluted and pooled at the final loading concentration of 70 pM.

### 2.5. Small RNA Sequencing and Data Analysis

Pooled libraries were sequenced on an Illumina iSeq™ 100 System (Illumina Inc, San Diego, CA, USA) with a run configuration of 1 × 51 bp, allowing approximately 2,000,000 raw reads per sample. All sequencing runs were performed with the same settings defined in Local Run Manager software version 2.4.0.2466. Secondary and tertiary analyses were performed using third-party software.

For quality control and identification of outlier samples, mirnaQC, a webserver for comparative quality control of miRNA-seq data, was used [26] in order to assess sequencing yield, sequencing quality, library complexity, and relative abundance of fragments from other RNA species. The mirnaQC tool generates output tables and visualizations that enable users to benchmark their results against a large reference dataset of similar samples. Instead of depending on arbitrary or user-defined thresholds that may lack justification, mirnaQC adopts a comparative approach. It calculates relative metrics based on a background distribution derived from over 36,000 miRNA-seq datasets spanning 25 species [26]. After a quality check, secondary and tertiary analyses were performed using the sRNAbench tool of the sRNA toolbox (https://arn.ugr.es/srnatoolbox/ (accessed on 1 December 2022)) [27]. Briefly, the FASTQ files were uploaded to the sRNAbench webserver for preprocessing and read alignment. miRBase release 22.1 was used as miRNA annotation reference database, while profiling was performed only for known human miRNAs by excluding other ncRNAs. Reads preprocessing was performed according to the SMARTer™ method recommendations, which included iterative trimming of the 5′ end of the read by means of iteratively aligning the reads to the genome sequence trimming unmapped reads by 1 nt (4 rounds of trimming). The adapter sequence to be detected was AAAAAAAAAAAA, while minimum length of adapter sequence to be aligned was 10. The number of allowed mismatches between adapter and reference sequences was 1. Alignment was performed using default parameters of the sRNAbench tool. Multidimensional scaling (MDS) analysis based on normalized count data generated within the DESeq2 workflow was performed in an unsupervised manner to assess global sample similarity and detect potential outliers. Samples demonstrating marked deviation from the overall clustering structure across the first principal dimensions were considered for exclusion prior to differential expression analysis. Outlier assessment was conducted prior to differential expression testing to ensure homogeneity of high-throughput sequencing data. In the current study, we employed the DeSeq2 method for identification of differentially expressed miRNAs (DEmiRNAs).

### 2.6. Selection of Candidate miRNA and mRNA Targets for Validation in Circulating EVs and PBMCs

The selection of candidate miRNAs for further validation in circulating EVs was performed using the bioinformatic interpretation of DEmiRNA targets coupled with literature research. miRNA–mRNA interaction networks were drawn with the miRNet 2.0 [28] online platform: https://www.mirnet.ca/ (accessed on 1 April 2025). The network was woven taking into account miRNAs known to be located in exosomes, using miRTarBase v8.0 as a resource of validated miRNA–gene interactions. To interpret the network interactions, enrichment analysis was performed on target genes, using the Kyoto Encyclopedia of Genes and Genomes (KEGG) database and hypergeometric test as the enrichment algorithm [28]. Molecular pathways were considered significantly enriched when adj. *p* < 0.01. Observed interactions and identified pathways related to macrophage polarization and immunomodulation were further evaluated using literature research. Complementary selected miRNA and mRNA candidates using the DEmiRNA network and the literature were further validated in circulating EVs and PBMCs, respectively.

### 2.7. Relative Expression of Candidate miRNAs in Circulating EVs and Target Genes in PBMCs

For quantification of relative miRNA levels, synthesis of complementary DNA (cDNA) was performed from total RNA in a reverse transcription reaction using a TaqMan^®^ microRNA Reverse Transcription kit and stem-loop primers, according to the manufacturer’s protocol (Thermo Fisher Scientific Inc., Waltham, MA, USA). To ensure the similar input amount of EV RNA, equal volumes of RNA extract were used. Additionally, a dilution of cel-miR-39 RNA was spiked in EV RNA samples during cDNA synthesis process to control for reverse transcription efficiency. The primer pool for reverse transcription consisted of TaqMan^®^ MicroRNA Assay reverse transcription primers at a final dilution of 0.05× for: cel-miR-39-3p (ID 000200), hsa-miR-98-5p (ID 000577), hsa-let-7i-5p (ID 002221), hsa-miR-19b-3p (ID 000396), hsa-miR-142-3p (ID 000464), hsa-miR-323b-3p (ID 244080_mat), and hsa-miR-16-5p (ID 000391). Synthesized cDNA was stored at −20 °C. The expression levels were determined by quantitative Real-time PCR on an Applied Biosystems Real-Time 7500 system (Applied Biosystems, Inc., Foster City, CA, USA) using the TaqMan microRNA assays for the corresponding miRNAs. hsa-miR-16-5p (ID 000391) was employed as endogenous reference gene for normalization of EV miRNA relative levels, based on its frequent use in circulating and extracellular vesicle miRNA studies and its consistent detectability across samples.

cDNA synthesis for relative quantification of target genes’ mRNA was performed using a RevertAid First strand cDNA synthesis kit according to the manufacturer’s protocol (Thermo Fisher Scientific Inc., Waltham, MA, USA) using 500 ng of total RNA. The mRNA levels of the selected target genes were determined by quantitative real-time PCR on an ABI 7500 Fast Real Time PCR System (Applied Biosystems, Inc., Foster City, CA, USA; Thermo Fisher Scientific Inc., Waltham, MA, USA) using TaqMan^®^ gene expression assays: Hs02621230_s1 for *PTEN* (phosphatase and tensin homolog), Hs00705164_s1 for *SOCS1* (suppressor of cytokine signaling 1), Hs02330328_s1 for *SOCS3* (suppressor of cytokine signaling 1), Hs00610320_m1 for *TGFBR1* (transforming growth factor beta receptor 1), and Hs00765553_m1 for *CCND1* (cyclin D1). Relative mRNA levels were normalized to a reference gene, *PPIA* (Peptidylprolyl Isomerase A), Hs04194521_s1. All reactions were performed in duplicate in a 96-optical well plate under the following conditions: 50 °C/2 min (1 cycle); 95 °C/10 min (1 cycle); and 95 °C/15 s, 60 °C/1 min (40 cycles).

### 2.8. Statistical Analysis of Relative miRNA and mRNA Expression

The relative levels of target miRNAs and mRNAs were calculated using the comparative Ct method [29]. The analysis of miRNA and mRNA relative levels between investigated sample groups was performed on 2^−dCt^ values. A Mann–Whitney U test was used to compare two unpaired groups of continuous variables that do not follow a normal distribution. Spearman’s rank correlation coefficient was used for testing the correlation between two variables, relative miRNA and gene expression (mRNA) levels. Values of *p* < 0.05 were considered statistically significant. All analyses and graphical presentation of results were performed using Prism v8 software (GraphPad Software, Inc., Boston, MA, USA).

## 3. Results

### 3.1. Study Population

After neuropathological analysis, IDH-wildtype glioblastoma was confirmed within 43 patients who were enrolled in the study. The discovery group in which the sequencing of the EV miRNome was performed included six patients and six age- and sex-matched healthy controls randomly selected from the whole cohort. The validation study of EV target miRNA and PBMC target mRNA expression enrolled 37 glioblastoma patients and 41 controls. Demographic characteristics of the investigated patients and controls are presented in the Table 1.

### 3.2. Characteristics of EVs Isolated from Plasma Samples

**Figure 1:**
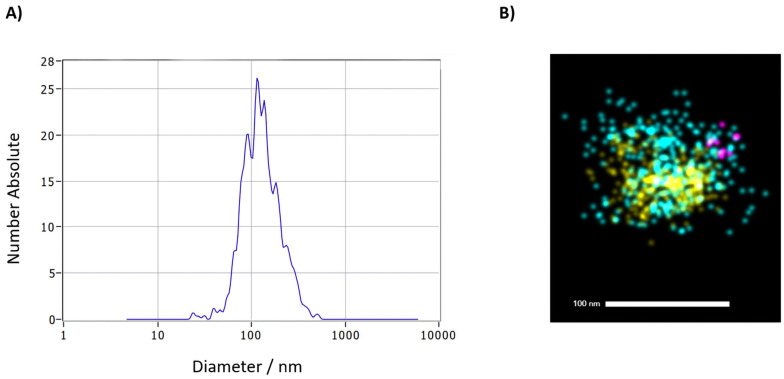
Confirmation of purified EVs. Representative images show (**A**) NTA analysis of a control sample displaying size distribution of non-labeled particles and (**B**) visualization of an EV from a control sample, using super-resolution microscopy. Tetraspanin markers are labeled with the following dyes: anti-CD9-488—cyan, anti-CD63-561—yellow, and anti-CD81-647—magenta.

The total number of particles in preparations was 8.32 × 10^10^ ± 1.59 × 10^10^ (mean ± SD), while their average size/hydrodynamic radius was 119.6 ± 6.6 nm (mean ± SD), as shown by NTA analysis of randomly selected control samples. All three analyzed tetraspanin EV markers, (CD81, CD63, and CD9) were confirmed by super-resolution microscopy, as shown on a representative EV (Figure 1), further validating the successful extraction of circulating EVs.

### 3.3. Differentially Expressed miRNAs in Circulating EVs of Glioblastoma Patients and Controls

Prior to DE analysis of small RNA sequencing data, a comprehensive quality control analysis was performed. Using the mirnaQC web tool, we did not identify technical discrepancies between study samples. Mean Phred Scores ranged from 34.8 to 35.4, which falls within the third quartile of the reference distribution, consistently above a substantial portion of the reference data. All of the samples had good library quality regarding the percentage of rRNA contamination (first quartile of reference data), depicting that libraries are rRNA purified, while for adapter dimers, one sample fell in first, eight samples in second, and three samples in the third quartile), the percentage of reads that correspond to adapter dimers, i.e., those that are shorter or equal to 2 nt after adapter trimming. By exploring library complexity, we found that ratio representing a number of identified miRNAs per 1000 reads falls in the first quartile for all samples. MDS visualization revealed one glioblastoma sample that showed clear separation from both the glioblastoma and control clusters across the first two principal dimensions. Based on this marked deviation in global expression structure, the sample was excluded from downstream differential expression analysis. The final discovery cohort therefore included five glioblastoma patients and six controls.

**Figure 2:**
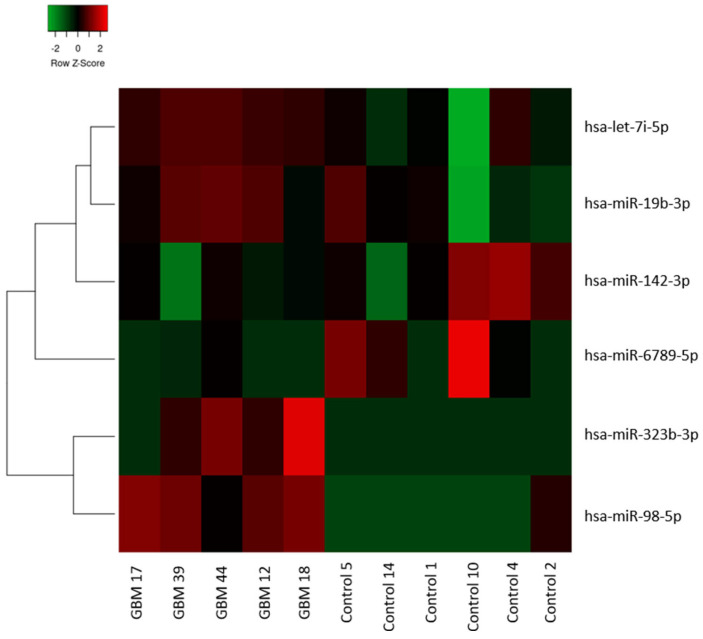
Heatmap of differentially expressed miRNAs. The heatmap represents the relative expression of DEmiRNAs across the samples and their co-expression clustering. Red color indicates overexpression, while green color shows downregulation of DEmiRNA levels in circulating EVs of glioblastoma patients compared to controls.

Based on stringent miRNA filtering strategies of the sRNAbench tool, a total of 53 identified miRNAs were enrolled in DE analysis. Of the identified miRNAs, five miRNAs were significantly differentially expressed in EVs between glioblastoma patients and controls according to the nominal *p* value. Of the DEmiRNAs, hsa-miR-6789-5p remained significantly differentially expressed after the FDR correction (Table 2). We additionally included hsa-miR-323b-3p as a sixth potential DEmiRNA, which has shown a trend toward differential expression and was also in a single co-expression branch with hsa-miR-98-5p (Figure 2), to be further explored in subsequent bioinformatic analysis and experimental validation.

### 3.4. Bioinformatic Analysis of DEmiRNAs Between Glioblastoma Patients and Controls and Selection of miRNA and mRNA Candidates for Experimental Validation

**Figure 3:**
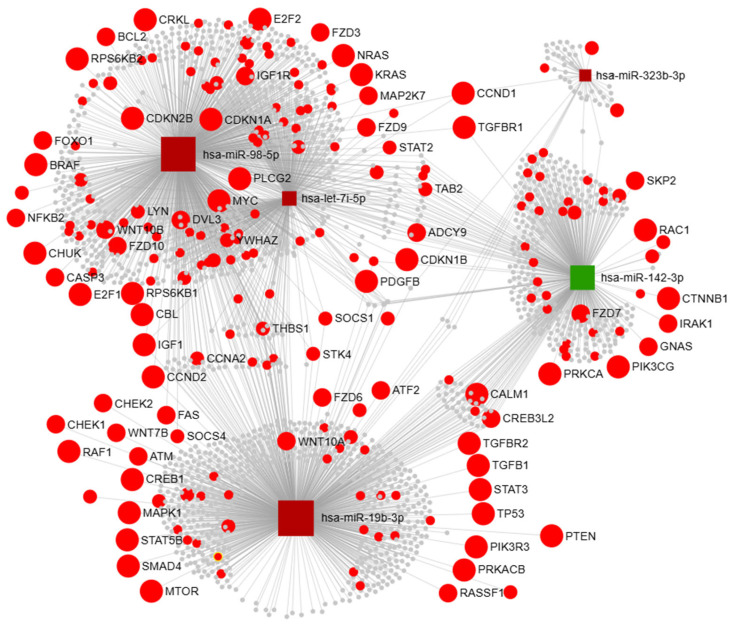
Integrated network of DEmiRNAs and their target genes. Green color of miRNA nodes (squares) represents downregulated miRNAs, while red color represents upregulated miRNAs in circulating EVs of glioblastoma patients compared to controls. Larger size of squares represents the higher number of interactions between target genes and corresponding miRNA. Circles represent target genes of network miRNAs. Red circles represent genes involved in the top significantly enriched KEGG pathways, while the size of the gene nodes (circles) increases if the gene is involved in multiple pathways and depicts the number of enriched pathways in which the gene is involved.

To visualize and prioritize the interactions of the hereby identified DEmiRNAs with their target genes, we employed miRNet 2.0 to generate an integrated network of DEmiRNA–target mRNA interactions (Figure 3). The subsequent enrichment analysis performed on the network genes has identified 23 enriched pathways (Table 3).

For validation analysis in circulating EVs, we chose all network miRNAs: hsa-miR-98-5p, hsa-let-7i-5p, hsa-miR-19b-3p, hsa-miR-142-3p, and hsa-miR-323b-3p. Although hsa-miR-6789-5p was the most significant DEmiRNA, there were no previously validated interactions for this miRNA that could be included in the network, and thus, the miRNA was omitted from the network by the algorithm. Additionally, only one study has investigated this miRNA so far [30], so there was not a sufficient body of literature supporting further validation of this miRNA in the context of the study aim.

For functional interpretation of the target genes collectively regulated by DEmiRNAs thus identifying key molecular pathways commonly regulated by the miRNAs from the integrated network, we performed KEGG pathway enrichment analysis of network genes. The majority of the obtained enriched pathways were directly involved in different malignancies (glioma, prostate cancer, colorectal cancer, bladder cancer, lung cancer, and acute and chronic myeloid leukemia), while a general term, pathways in cancer, was the top enriched pathway in network genes. Additionally, pathways implicated in carcinogenesis (p53 signaling pathway, cell cycle, TGF-beta signaling, ErbB signaling pathway, Jak-STAT signaling, neurotrophin signaling, and insulin signaling) were also enriched in the network genes.

For further gene expression validation in PBMCs, we selected *SOCS1*, *SOCS3*, *PTEN*, *TGFBR1,* and *CCND1*. These targets were chosen because they represent major network hubs involved in the top significantly enriched KEGG pathways and experimentally confirmed or strongly predicted downstream effectors of the EV miRNAs identified in our study. Both *SOCS1* and *SOCS3* are well-established regulators of the JAK/STAT3 pathway, frequently downregulated in glioblastoma and other cancers [31,32,33] and directly targeted by hsa-miR-19b-3p [34,35,36] and hsa-miR-98-5p [37]. *PTEN*, another validated target of miR-19b-3p, is a negative regulator of the PI3K/AKT pathway and is critical for macrophage polarization [38,39]. TGFBR1 was included due to its previously reported responsiveness to miR-142-3p modulation in monocyte/macrophage subsets [40] and complementary network regulation by hsa-miR-98-5p. Finally, *CCND1* was selected as a network hub regulated by hsa-miR-98-5p, hsa-let-7i-5p, and hsa-miR-323b-3p, with prior evidence linking its dysregulation to immunosuppressive cell recruitment in the tumor microenvironment [41]. Collectively, these genes represent biologically relevant nodes in pathways of immune modulation, macrophage polarization, and tumor–immune crosstalk, providing a mechanistic rationale for their validation in an independent cohort.

### 3.5. Relative Expression of Circulating EV DEmiRNAs and PBMC Target Genes in Glioblastoma Patients and Controls

**Figure 4:**
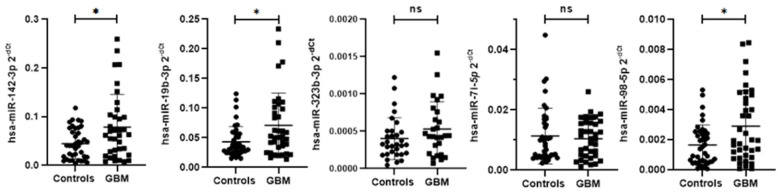
The difference in relative circulating EV miRNA levels between glioblastoma patients and controls. Relative miRNA levels were standardized against the hsa-miR-16 endogenous control and presented as a scatterplot of 2^−dCt^ values with standard errors of the mean. Differences in relative miRNA levels of targeted circulating EV miRNAs were analyzed using the Mann–Whitney U test: hsa-miR-142-3p, *p* = 0.036; hsa-miR-323b-3p, *p* = 0.17; hsa-miR-19b-3p, *p* = 0.026; hsa-let-7i-5p, *p* = 0.89; and hsa-miR-98-5p, *p* = 0.033. GBM—patients with glioblastoma. *—significant difference at *p* < 0.05; ns—nonsignificant difference in relative target gene mRNA levels.

**Figure 5:**
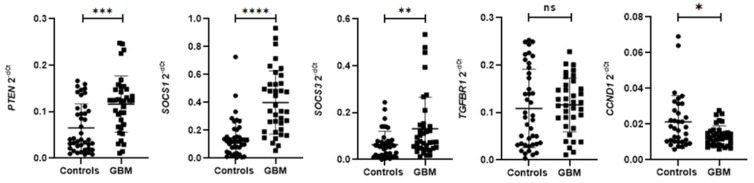
Difference in relative PBMC mRNA levels between glioblastoma patients and controls. Relative mRNA levels were standardized against the endogenous control *PPIA* mRNA level and presented as a scatterplot of 2^−dCt^ values with standard errors of the mean. Differences in relative mRNA levels of targeted genes were analyzed using the Mann–Whitney U test: *PTEN* mRNA, *p* = 0.0002; *SOCS1* mRNA, *p* < 0.0001; *SOCS3* mRNA, *p* = 0.0129; *TGFBR1* mRNA, *p* = 0.3287; and *CCND1* mRNA, *p* = 0.0191. GBM—patients with glioblastoma. * significant difference at *p* < 0.05; ** significant difference at *p* < 0.01; *** significant difference at *p* < 0.001; **** significant difference at *p* < 0.0001; ns—non-significant difference in relative target gene mRNA levels.

The validation analysis of relative expression of the identified DEmiRNAs has shown a statistically significant upregulation of hsa-miR-142-3p, hsa-miR-19b-3p, and hsa-miR-98-5p in circulating EVs of glioblastoma patients compared to controls (Table 4, Figure 4). By profiling relative mRNA expression from the same subjects, we identified statistically significant upregulation of *PTEN*, *SOCS1,* and *SOCS3* in glioblastoma patients compared to controls, while *CCND1* was significantly downregulated in glioblastoma patients compared to controls (Table 4, Figure 5).

Correlation analysis between the relative mRNA expression levels of target genes and the corresponding DEmiRNAs in glioblastoma patients revealed certain significant positive associations. *PTEN* expression positively correlated with hsa-miR-142-3p, hsa-miR-323b-3p, and hsa-miR-98-5p (Table 5). Additionally, *TGFBR1* expression showed a positive correlation with hsa-miR-323b-3p, while CCND1 expression was positively correlated with hsa-miR-98-5p (Table 5). In contrast, no significant correlations were observed between the expression levels of target genes and the corresponding DEmiRNAs in the control group (Table 5).

## 4. Discussion

By profiling both plasma-derived EV miRNAs and PBMC target gene expression in matched glioblastoma and control samples, this study captures a cross-sectional view of peripheral molecular alterations in the circulation of glioblastoma patients, potentially compatible with early systemic immune adaptations related to monocyte/macrophage polarization-associated pathways.

The two miRNAs that were consistently significantly upregulated in circulating EVs both in miRNAseq and in the validation experiment were hsa-miR-19b-3p and hsa-miR-98-5p. Similarly to our study, hsa-miR-19b overexpression has been also identified in glioma tissue and cell lines [42], and its expression been shown to positively correlate with the glioma tumor grade [42,43]. Furthermore, inhibition of miR-19 by anti-miR-19 resulted in diminished proliferation of U251MG glioblastoma cells in vitro [44]. In like manner, hsa-miR-98 was also identified to be dysregulated in different malignancies including glioblastoma but with a multifaceted role in cancer progression, exhibiting both oncogenic and tumor-suppressive properties, depending on the cancer context and microenvironment [45,46]. It was shown that exosomal miR-19b-3p from the plasma of esophageal squamous cell carcinoma (ESCC) patients promotes tumorigenesis by suppressing MAP2K3 [47]. Since MAP2K3 suppress STAT3 expression, its inhibition results in elevated STAT3 levels, which subsequently upregulate miR-19b-3p expression through direct promoter binding, establishing a regulatory feedback loop in ESCC [47]. STAT3 plays a pivotal role in macrophage polarization, serving as a convergence point for various signaling pathways. JAK/STAT, one of the top enriched pathways in our study, is a main signaling pathway for the activation of more than 70 cytokines, and it plays an important role in numerous crucial biological processes such as cell proliferation, differentiation, apoptosis, and immune regulation [48,49]. In malignancies such as glioma and ovarian cancer, activation of the JAK/STAT3 signaling pathway has been shown to promote M2 macrophage polarization and influence disease progression by modulating specific cytokines [50,51]. The SOCS protein family represents the most prominent group of inducible inhibitors within the JAK/STAT3 pathway, with a key role in promoting M1 polarization [52,53]. Given their critical inhibitory function, dysregulation of SOCS1 and SOCS3 may provide an additional link between miR-19b-3p activity and persistent JAK/STAT3 activation. Glioma tissue levels of both SOCS1 and SOCS3 have been shown to be downregulated [31,32]. SOCS1 exerts a strong antiproliferative effect on tumor cells and is dependent on the inhibition of STAT3 and on other signaling proteins [54]. *SOCS3* is often epigenetically silenced in cancer, contributing to uncontrolled tumor cell growth. Like SOCS1, SOCS3 markedly reduces the expression of cell cycle regulatory proteins CDK2, CDK4, cyclin E, and cyclin D1 in prostate cancer [55]. In hepatocellular carcinoma, methylation-mediated silencing of SOCS3 enhances IL-6/JAK/STAT3 signaling and FAK phosphorylation [56]. Both SOCS1 and SOCS3 have been identified as direct targets of hsa-miR-19b-3p [34,35,36], whereas to date, hsa-miR-98-5p has been reported to target only SOCS1 [37].

Previously reported downregulation of SOCS1 and SOCS3 expression in glioblastoma tumor tissue [32,33] aligns with the increased levels of hsa-miR-19b-3p and hsa-miR-98-5p observed in circulating EVs of glioblastoma patients in this study, both of which are known to post-transcriptionally regulate SOCS gene expression. However, in the present study, we observed a significant upregulation of both *SOCS1* and *SOCS3* in PBMCs of glioblastoma patients compared to healthy controls. Despite the known regulatory interaction between these miRNAs and SOCS genes, we did not detect any significant correlation between *SOCS1* or *SOCS3* expression levels in PBMCs and circulating EV miRNA abundance, suggesting that complex and indirect regulatory mechanisms may be more influential in the peripheral compartment compared to events observed in the tumor tissue. Methylation of promotors of SOCS genes is the most prominent mechanism of their mRNA expression downregulation in the tumor, as it was described that in methylated glioblastoma, mRNA expression for SOCS1-2-3 was reduced when compared with unmethylated glioblastoma. Moreover, methylation of *SOCS3* promoter significantly associated with an unfavorable clinical outcome [31]. However, the effects observed in the peripheral PBMCs could be a result of different pattern of stimuli. While SOCS1 has been shown to modulate and restrain M1-driven inflammation, it is also upregulated during M2 macrophage polarization, supporting anti-inflammatory functions. Conversely, SOCS3 is generally associated with M1-like inflammatory macrophages, inhibiting STAT3 and PI3K signaling to maintain a pro-inflammatory state [53]. Therefore, the upregulation of both *SOCS1* and *SOCS3* in PBMCs could indicate a primed, inflammatory monocyte population that remains responsive to systemic tumor-associated stimuli but has not yet undergone full phenotypic reprogramming into immunosuppressive TAMs. It is already known that in early tumor development, the glioblastoma microenvironment is characterized by pro-inflammatory cytokines that induce M1-like tumor-associated macrophages, which initially exert anti-tumor effects but later promote tumor progression through chronic inflammation and genomic instability [57,58]. As the tumor progresses, TAMs are reprogrammed toward an M2-like immunosuppressive phenotype, supporting angiogenesis, immune evasion, and extracellular matrix remodeling [59,60]. Altogether, these findings are compatible with a complex and dynamically regulated systemic immune response in glioblastoma, where SOCS1-mediated anti-inflammatory feedback may arise to counterbalance SOCS3-driven inflammatory signaling, potentially preparing circulating monocytes for subsequent polarization and functional adaptation upon tumor infiltration. This supports the emerging view of macrophage polarization as a dynamic and transitional continuum, rather than a strict M1/M2 dichotomy, especially within cancer-associated contexts [61].

PTEN is a well-established negative regulator of the PI3Kγ signaling pathway [38] and plays important role in macrophage polarization [62]. Based on the observed upregulation of hsa-miR-19b-3p in EVs from glioblastoma patients and its known role in downregulating PTEN, an event known to promote M2-like polarization [39], a reduced *PTEN* expression in PBMCs was anticipated. However, in contrast to this expectation, we found *PTEN* expression to be significantly upregulated in PBMCs of glioblastoma patients. This finding mirrors the pattern observed for SOCS1 and SOCS3, further supporting the interpretation that peripheral monocytes in glioblastoma may exhibit a transcriptional profile consistent with pro-inflammatory activation, potentially influenced by circulating tumor-associated cues, without evidence of complete immunosuppressive programming. Interestingly, although *PTEN* is not a known direct target of hsa-miR-98-5p, we observed a significant positive correlation between *PTEN* and hsa-miR-98-5p expression. Supporting this observation, overexpression of miR-98 in hypopharyngeal carcinoma cells was previously shown to increase PTEN expression by directly targeting MTDH, thus inducing apoptosis by regulating the PTEN/AKT/caspase-3/9 pathway via MTDH [63]. Taken together, the concurrent upregulation of hsa-miR-98-5p, *PTEN*, *SOCS1*, and *SOCS3* in PBMCs implies a transitional immune phenotype, where in pro-inflammatory signaling predominates but is being modulated by regulatory feedback loops, potentially preparing circulating monocytes for later adaptation into tumor-associated macrophages upon recruitment into the TME.

CCND1 was also identified as a network hub regulated by multiple miRNAs, with particular interest in hsa-miR-98-5p, which was previously confirmed to be upregulated in EVs from glioblastoma patients in an independent cohort. A prior study demonstrated that CCND1 amplification in melanoma patients was associated with a higher proportion of immunosuppressive cells (Treg cells and M2 macrophages) and a lower proportion of immune boosting cells (follicular helper T cells, naïve B cells, and CD8+ T cells) in the TME [41]. In the present study, the observed downregulation of *CCND1* in PBMCs aligns with the upregulation of circulating EV hsa-miR-98-5p. Although direct evidence for CCND1 as a determinant of macrophage phenotype is lacking, its upregulation has been linked to the preferential recruitment of M2-polarized macrophages to the tumor microenvironment. This relationship raises the possibility that *CCND1* downregulation in PBMCs of glioblastoma patients may contribute to peripheral immune modulation, potentially favoring pro-inflammatory mechanisms in circulation, which has to be investigated further.

Although small RNA-seq screening in EVs suggested downregulation of hsa-miR-142-3p in glioblastoma patients compared to controls, validation on an independent, larger cohort has pointed to upregulation of this miRNA in glioblastoma patients compared to controls. Discordances between discovery RNA-seq and subsequent qPCR validation, including direction reversals for some miRNAs, have been reported in original studies and cross-platform evaluations, particularly for low-input RNA material such as exosomal RNA [64]. Although cautiously, we will interpret miR-142-3p as upregulated in EVs of glioblastoma patients as observed in the qPCR experiment. In a previous study, investigating expression profiles between human glioblastoma-infiltrating macrophages and matched peripheral monocytes, miR-142-3p was the most downregulated miRNA (approximately 4.95-fold) in glioblastoma-infiltrating macrophages [40]. Although the previous study did not find statistically significant differences in miR-142-3p levels in peripheral monocytes from patients with glioblastoma compared to healthy controls [40], the levels of this miRNA were found to be higher in M1 macrophages than in M2, which further suggests the existence of pro-inflammatory cues in circulating EVs in which miR-142-3p levels were increased in glioblastoma patients compared to controls. Additionally, it was described that the levels of TGFBR1 were responsive only in M2 macrophages after anti-miR-142-3p treatment [40], suggesting that this contextual and cell-specific effect aligns more on the M1 part of the scale in our study, as TGFBR1 was not found to be differentially expressed in PBMCs of glioblastoma patient in our study. Subsequent studies should further investigate EV miR-142-3p miRNA levels and their ability to influence monocyte gene expression prior to extravasation toward a tumor, allowing possibilities for therapeutic applications.

Several factors limit the interpretation of the present findings. While our study integrates matched EV miRNA and PBMC gene expression profiles, it remains cross-sectional. Thus, temporal dynamics of immune modulation during glioblastoma progression cannot be inferred. Additionally, the EV miRNAs detected may originate from multiple cellular sources, including immune and stromal cells, not exclusively glioblastoma cells, complicating attribution of observed effects to tumor-derived vesicles. However, glioblastoma-derived EVs have been shown to modulate the circulating myeloid cell phenotype through RNA transfer [65], reinforcing the biological relevance of investigating EV miRNA–PBMC gene expression correlations as potential indicators of early systemic immune modulation. EV-enriched RNA was isolated using a commercial resin-based purification system. Although the manufacturer states that purified exosomes are free of contaminating RNA-binding proteins, independent characterization of vesicle purity was not performed in this study, thus not excluding non-vesicular circulating components. Additionally, molecular heterogeneity of glioblastoma may limit generalizability, particularly in light of inter-patient differences in tumor subtype, treatment status, and systemic inflammation. Therefore, given the limited size of the discovery cohort, additional high-throughput profiling of EV miRNA expression in GBM patients, taking into account these stratification parameters, is warranted to provide deeper insight into the EV miRNome in GBM. Nevertheless, the patient cohort employed herein was appropriate for hypothesis generation, bioinformatic analysis, and the selection of candidate miRNAs and mRNAs for further validation on a larger group.

## 5. Conclusions

This study provides evidence that circulating EV miRNA cargo in glioblastoma patients is associated with distinct gene expression signatures in peripheral immune cells, suggesting an early, transcriptionally responsive immune state. The concurrent upregulation of hsa-miR-19b-3p, hsa-miR-98-5p, and hsa-miR-142-3p in circulating EVs and *SOCS1*, *SOCS3*, and *PTEN* in PBMCs points to a complex regulatory balance between pro-inflammatory and anti-inflammatory cues, potentially preceding full tumor-associated macrophage polarization. These findings support the concept that systemic immune alterations observed in glioblastoma are detectable in the peripheral circulation, where circulating EV-associated miRNAs are linked to coordinated gene expression patterns in immune cells. Future longitudinal and mechanistic studies are warranted to determine whether these circulating molecular signatures can serve as early biomarkers or therapeutic targets in glioblastoma immunomodulation.

## Figures and Tables

**Table 1:** Demographic characteristics of the studied group.

	Sequencing Cohort			Validation Cohort		
Variable	Controls (n = 6)	Glioblastoma (n = 6)	*p*	Controls (n = 41)	Glioblastoma (n = 37)	*p*
**sex, N (%)**						
**male**	4 (66.7)	4 (66.7)	1.00 ^a^	22 (53.7)	20 (54)	0.97 ^a^
**female**	2 (33.3)	2 (33.3)		19 (46.3)	17 (46)	
**age, years**	39.5 (32–54)	41 (33–55)	0.84 ^b^	58 (31–76)	64 (33–76)	0.7 ^b^

Statistical tests used for analysis of variables between two study groups: ^a^ Chi-squared and ^b^ Mann–Whitney U tests; *p* value less than 0.05 was considered statistically significant. Age is presented as the median with the minimum and maximum range.

**Table 2:** Differentially expressed miRNAs in circulating EVs of glioblastoma patients and controls.

DEmiRNA	log2FoldChange	*p*	Padj
hsa-miR-6789-5p	−6.639430931	0.000449991	0.02385
hsa-miR-98-5p	2.675771568	0.008265713	0.219041
hsa-let-7i-5p	0.909725899	0.018148472	0.320623
hsa-miR-19b-3p	0.987763714	0.042660684	0.487453
hsa-miR-142-3p	−1.066905218	0.045986135	0.487453
hsa-miR-323b-3p	2.229767902	0.065195964	0.575898

log2FoldChange—indicates the gene expression changes in circulating EVs of glioblastoma patients compared to control samples on a logarithmic scale to base 2; *p*—Wald test *p* value of the DeSeq2 algorithm; Padj—*p* value adjusted for multiple testing with the Benjamini–Hochberg procedure, which controls the false discovery rate (FDR).

**Table 3:** KEGG pathway enrichment analysis of the DEmiRNA target genes from the integrated network.

Name	Hits	Pval	adj.Pval
Pathways in cancer	68	1.92 × 10^−8^	1.92 × 10^−6^
Prostate cancer	25	6.07 × 10^−6^	0.000178
Chronic myeloid leukemia	22	9.49 × 10^−6^	0.000178
p53 signaling pathway	21	9.92 × 10^−6^	0.000178
TGF-beta signaling pathway	24	1.04 × 10^−5^	0.000178
Cell cycle	31	1.16 × 10^−5^	0.000178
Colorectal cancer	17	1.26 × 10^−5^	0.000178
HTLV-I infection	43	1.42 × 10^−5^	0.000178
Glioma	20	1.72 × 10^−5^	0.000191
ErbB signaling pathway	24	0.00002	0.0002
Jak-STAT signaling pathway	25	6.86 × 10^−5^	0.000624
Melanogenesis	25	9.76 × 10^−5^	0.000813
Alcoholism	35	0.000153	0.00118
Bladder cancer	11	0.000178	0.00127
Measles	24	0.000309	0.00206
Epstein–Barr virus infection	22	0.000364	0.00228
Non-small cell lung cancer	15	0.000428	0.00252
Vibrio cholerae infection	8	0.000614	0.00341
Neurotrophin signaling pathway	26	0.00103	0.00542
Insulin signaling pathway	28	0.00116	0.0058
Acute myeloid leukemia	15	0.00123	0.00586
Pancreatic cancer	17	0.00133	0.00605
Fc gamma R-mediated phagocytosis	21	0.00224	0.00974

Hits—number of genes in the network involved in the pathway; *p*—hypergeometric test *p* value; Padj—adjustment for false discovery rate (FDR).

**Table 4:** Difference in relative DEmiRNA and target gene levels between glioblastoma patients and controls.

	Fold Change	*p*
**Circulating EV miRNA**		
**hsa-miR-142-3p**	*1.76*	*0.04*
**hsa-miR-19b-3p**	*1.64*	*0.03*
**hsa-miR-323b-3p**	0.80	0.17
**hsa-let-7i-5p**	0.93	0.89
**hsa-miR-98-5p**	*1.81*	*0.03*
		
**PBMC target gene mRNA**		
***PTEN***	*1.57*	*0.0002*
***SOCS1***	*2.76*	*<0.0001*
***SOCS3***	*1.92*	*0.0129*
***TGFBR1***	1.16	0.3287
***CCND1***	*0.63*	*0.0191*

A Mann–Whitney U test was used for the analysis of difference between the study groups. Fold change indicates DEmiRNA and gene expression changes in glioblastoma patients compared to controls. *p* < 0.05 denotes statistically significant differential expression, which is marked in italic.

**Table 5:** Correlation analysis.

Variable		Controls		GBM	
		R	*p*	r	*p*
**hsa-miR-142-3p**					
	*PTEN* mRNA	−0.04	0.79	*0.38*	*0.02*
	*SOCS1* mRNA	0.19	0.24	−0.08	0.64
	*SOCS3* mRNA	−0.02	0.90	0.09	0.61
	*TGFBR1* mRNA	0.01	0.95	0.30	0.07
	*CCND1* mRNA	0.10	0.60	0.09	0.62
**hsa-miR-323b-3p**					
	*PTEN* mRNA	0.02	0.90	*0.40*	*0.04*
	*SOCS1* mRNA	−0.04	0.83	−0.01	0.95
	*SOCS3* mRNA	−0.03	0.85	0.32	0.10
	*TGFBR1* mRNA	−0.09	0.62	*0.40*	*0.04*
	*CCND1* mRNA	0.08	0.69	−0.32	0.10
**hsa-miR-19b-3p**					
	*PTEN* mRNA	−0.04	0.82	0.30	0.07
	*SOCS1* mRNA	0.18	0.26	0.04	0.82
	*SOCS3* mRNA	−0.04	0.81	0.10	0.57
	*TGFBR1* mRNA	0.01	0.96	0.07	0.67
	*CCND1* mRNA	0.09	0.60	0.14	0.41
**hsa-let-7i-5p**					
	*PTEN* mRNA	−0.001	0.99	0.24	0.16
	*SOCS1* mRNA	−0.03	0.87	−0.03	0.88
	*SOCS3* mRNA	−0.04	0.78	0.07	0.68
	*TGFBR1* mRNA	−0.007	0.96	0.12	0.48
	*CCND1* mRNA	−0.0003	0.99	0.06	0.77
**hsa-miR-98-5p**					
	*PTEN* mRNA	0.12	0.46	*0.46*	*0.004*
	*SOCS1* mRNA	0.11	0.51	−0.17	0.32
	*SOCS3* mRNA	0.04	0.79	0.12	0.49
	*TGFBR1* mRNA	0.05	0.77	*0.38*	*0.02*
	*CCND1* mRNA	0.06	0.74	0.02	0.88

Spearman’s rank correlation coefficient was used for testing the correlation between two variables, relative miRNA and gene expression (mRNA) levels. *p* < 0.05 denotes statistically significant correlation, which is marked in italic.

## Data Availability

The datasets supporting the conclusions of this article are included within the article. Raw sequencing data will be available upon reasonable request.
